# Naphthoquine: A Potent Broad-Spectrum Anti-Coronavirus Drug In Vitro

**DOI:** 10.3390/molecules27030712

**Published:** 2022-01-21

**Authors:** Yabin Song, Yongqiang Deng, Huiqiang Wang, Zhuchun Bei, Hongjing Gu, Hui Zhao, Hong Wang, Dongna Zhang, Likun Xu, Baogang Wang, Yuhuan Li, Hongquan Wang

**Affiliations:** 1 State Key Laboratory of Pathogen and Biosecurity, Beijing Institute of Microbiology and Epidemiology, Beijing 100071, China; songyb@126.com (Y.S.); dengyq1977@126.com (Y.D.); beizhuchun@hotmail.com (Z.B.); ghj0048@163.com (H.G.); shuishu2002@126.com (H.Z.); ammswh@126.com (H.W.); donna_1117@163.com (D.Z.); likun_xu@163.com (L.X.); wbg0423@163.com (B.W.); 2 CAMS Key Laboratory of Antiviral Drug Research, Beijing Key Laboratory of Antimicrobial Agents, Institute of Medicinal Biotechnology, Chinese Academy of Medical Sciences and Peking Union Medical College, Beijing 100050, China; hq_wangimb@163.com

**Keywords:** coronavirus, SARS-CoV-2, repurposing, malaria, Naphthoquine

## Abstract

COVID-19 has spread around the world and caused serious public health and social problems. Although several vaccines have been authorized for emergency use, new effective antiviral drugs are still needed. Some repurposed drugs including Chloroquine, Hydroxychloroquine and Remdesivir were immediately used to treat COVID-19 after the pandemic. However, the therapeutic effects of these drugs have not been fully demonstrated in clinical studies. In this paper, we found an antimalarial drug, Naphthoquine, showed good broad-spectrum anti-coronavirus activity. Naphthoquineinhibited HCoV-229E, HCoV-OC43 and SARS-CoV-2 replication in vitro, with IC_50_ = 2.05 ± 1.44 μM, 5.83 ± 0.74 μM, and 2.01 ± 0.38 µM, respectively. Time-of-addition assay was also performed to explore at which stage Naphthoquine functions during SARS-CoV-2 replication. The results suggested that Naphthoquine may influence virus entry and post-entry replication. Considering the safety of Naphthoquine was even better than that of Chloroquine, we think Naphthoquine has the potential to be used as a broad-spectrum drug for coronavirus infection.

## 1. Introduction

Coronaviruses (order Nidovirales, family Coronaviridae, genus Coronavirus) are enveloped positive-sense single-stranded RNA viruses that are widely found in nature [1,2]. Of the seven known coronavirus species that infect humans, four are more common in humans (HCoV-229E, HCoV-OC43, HCoV-NL63 and HCoV-HKU1), which are less pathogenic and generally cause only mild respiratory symptoms resembling the common cold [1,2]. Three other coronaviruses pose serious threats to human health, including severe acute respiratory syndrome coronavirus (SARS-CoV), Middle East respiratory syndrome coronavirus (MERS-CoV), and novel coronavirus (SARS-CoV-2) [3,4]. The disease caused by SARS-CoV-2 was named COVID-19 [5].

At present, COVID-19 has spread around the world. It has become the most serious infectious disease epidemic since the Spanish flu broke out in 1918. As of 6 December 2021, a total of 265,194,191 cases and 5,254,116 deaths have been confirmed globally, and the number of confirmed and deaths is increasing [6]. Because SARS-CoV-2 is highly infectious and deadly, it poses a great threat to human health and social stability. Although several vaccines have been authorized for emergency use and are being deployed, it will still take a long time to achieve herd immunity globally [7,8,9]. In addition, the emergence of mutated viruses reduces the effectiveness of vaccines. Therefore, new effective antiviral drugs are still needed [10]. Moreover, broad-spectrum antivirals can be used for other coronaviruses that might emerge in the future. It is well known that a traditional new drug discovery always takes several years; therefore, the drug repurposing method was immediately used to explore treatments for COVID-19 after the pandemic began. It is a strategy of identifying the approved and investigational drugs for new uses that are authorized for the treatment of other diseases [11,12]. As approved drugs have sufficient data on pharmacokinetics, pharmacodynamics, and toxicity, they can be rapidly brought into clinical studies [11,12]. In fact, some approved drugs have shown high efficacy against SARS-CoV-2 in vitro, such as lopinavir and ritonavir, chloroquine (CQ), favipiravir, and remdesivir (RDV) [13]. However, the therapeutic effects of most drugs have not been fully demonstrated in clinical studies [14]. 

Studies have found that some quinoline antimalarials have good antiviral activities, such as CQ, hydroxychloroquine (HCQ), and mefloquine [15,16,17]. They all showed good anti-SARS-CoV-2 activity in vitro with IC_50_ value less than 10 μM [15,17]. In particular, CQ and HCQ were selected as candidates and approved for urgent use in the clinical treatment of COVID-19 [18]. However, lethal side effects, such as hypoglycaemia and prolongation of the QTc interval, raised safety concerns [19]. In addition, the results of these clinical studies were often non-significant [20,21,22]. Therefore, the FDA withdrew emergency use of CQ and HCQ for the treatment of COVID-19 in June 2020.

**Figure 1:**
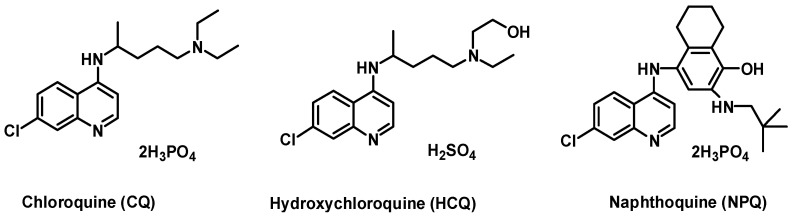
Selected 4-aminoquinoline antimalarial drugs.

Naphthoquine (NPQ) is a 4-aminoquinoline antimalarial synthesized for the first time in our lab in China in 1986 (Figure 1) [23]. Since 2003, a single-dose fixed co-formulations of NPQ and artemisinin has been marketed under the name “ARCO” [23]. Four thousand healthy volunteers and malaria patients were exposed to NPQ without any recorded significant toxicity [23]. No cardiac or neurological events have been reported. Based on the superior efficacy and safety data of therapies containing NPQ in existing studies, NPQ has been proposed as a new candidate for antimalarial treatment and prophylaxis [24]. Due to its similar structure to CQ, NPQ may have good anti-coronavirus activity.

In this study, we found that NPQ had good antiviral activity against HCoV-OC43, HCoV-229E and SARS-CoV-2 in vitro. The preclinical data obtained by us on NPQ will provide some scientific basis for further studies in humans with the molecule.

## 2. Results

### 2.1. NPQ Inhibits HCoV-229E and HCoV-OC43 Replication In Vitro

**Figure 2:**
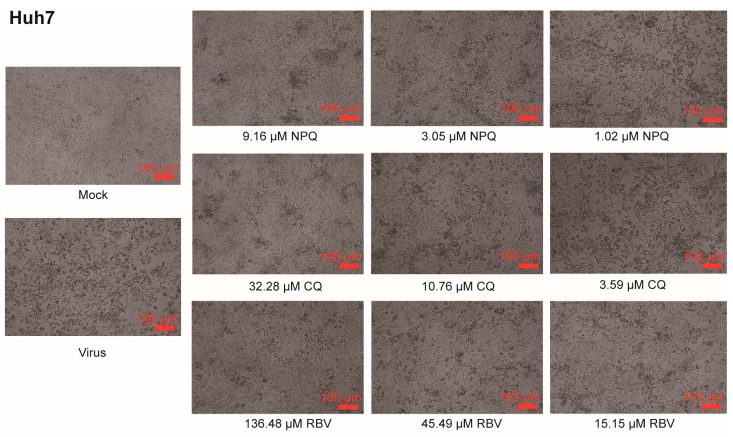
NPQ, CQ and RBV all dose-dependently inhibit CPE induced by HCoV-229E infection. Cells were mock infected or infected with virus in the presence of NPQ, CQ and RBV. Virus-produced CPE was observed by microscopy at 48 h post infection.

In this study, cytopathic effect (CPE) assay was used to detect the toxicity of NPQ and other drugs in cells, and their inhibitory activity against coronaviruses HCoV-OC43 and HCoV-229E. Ribavirin (RBV) was used as a positive control. As shown in Figure 2, compared to the untreated controls (virus), in which almost all cells became rounded and refractile resulting in a subtotal destruction of the cell monolayer, NPQ, CQ and RBV can all dose-dependently inhibit the cytopathic effects induced by HCoV-229E infection in Huh7 cells. The 50% toxicity concentration (CC_50_) of NPQ was 11.50 ± 6.19 μM, the IC_50_ against HCoV-229E was 2.05 ± 1.44 μM, and the selection index (SI) was 5.61, which was comparable to CQ (Table 1).

**Figure 3:**
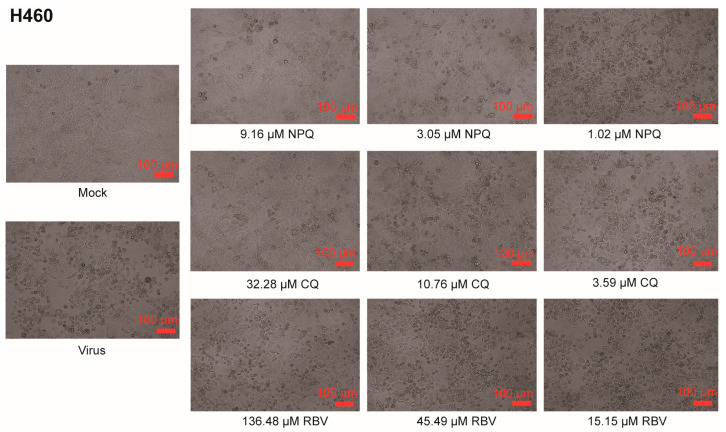
NPQ, CQ and RBV all dose-dependently inhibit CPE induced by HCoV-OC43 infection. Cells were mock infected or infected with virus in the presence of NPQ, CQ and RBV. Virus-produced CPE was observed by microscopy at 72 h post infection.

As shown in Figure 3, NPQ, CQ and RBV can all dose-dependently inhibit the cytopathic effects induced by HCoV-OC43 infection in H460 cells. The CC_50_ of NPQ was >82.52 μM and the IC_50_ against HCoV-OC43 was 5.83 ± 0.74 μM, and the selection index was >14.15, which was comparable to CQ and RBV (Table 2).

### 2.2. NPQ Inhibits SARS-CoV-2 Replication In Vitro

**Figure 4:**
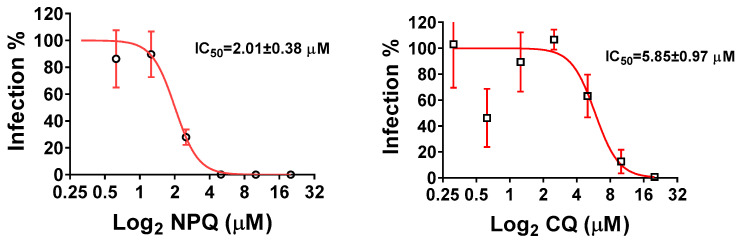
Dose-dependent inhibition of SARS-CoV-2 infection by addition of NPQand CQ in Vero cells.

Since NPQ can inhibit the replication of HCoV-OC43 and HCoV-229E, it may also inhibit SARS-CoV-2 replication. Viral RNA copies in the supernatants were determined by quantitative real-time PCR (qRT-PCR) to determine the antiviral effects of the compounds. The results showed that NPQ had an inhibitory effect on SARS-CoV-2, with IC_50_ of 2.01 ± 0.38 µM (Figure 4). While the IC_50_ of CQ is 5.85 ± 0.97 µM. The selection index of NPQ and CQ were 6.72 and 13.09, respectively. These results indicate that the inhibitory effect of NPQ on SARS-CoV-2 is similar to that of CQ (Table 3).

### 2.3. Time-of-Addition Analysis of NPQ against SARS-CoV-2

**Figure 5:**
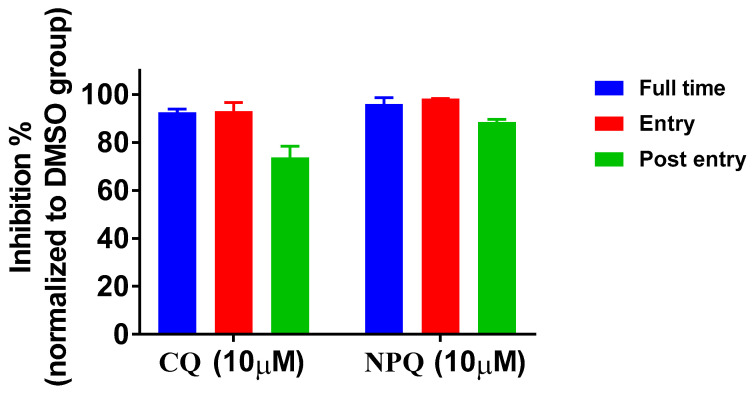
Time-of-addition assay of NPQ and CQ against SARS-CoV-2.

Time-of-addition assay was performed to explore at which stage NPQ functions during SARS-CoV-2 replication [17,25]. NPQ and CQ were treated at three different time points, including the whole experiment (full-time), the first 3 h and the last 46 h. The results showed that NPQ and CQ could also effectively inhibit SARS-CoV-2 infection at all time points (Figure 5). Especially NPQ remarkably reduced viral RNA levels to under 10% when applied at full time and the entry phase. These results suggest that NPQ may influence virus entry and post-entry replication.

## 3. Discussion

CQ and HCQ have been expected to be effective as anti-SARS-CoV-2 drugs given their good in vitro activity. However, neither lab-based studies nor clinical trials have provided consistent evidence to support their therapeutic value in the treatment of COVID-19 [20,21]. Developing new treatments of COVID-19 is still needed. In this study, CQ was chosen as a reference drug. The IC_50_ for CQ against HCoV-229E, HCoV-OC43 and SARS-CoV-2 calculated in our study were 8.74 ± 7.27, 3.16 ± 0.60 and 5.85 ± 0.97 µM, respectively, which were consistent with the IC_50_ values at µM ranges examined in other studies [15,26]. It was reported that the sensitivity to antivirals, such as CQ and HCQ, depended on cell types [17,27], so Huh7, H460 and Vero cells were used for HCoV-229E, HCoV-OC43 and SARS-CoV-2 virus infection, respectively.

NPQ and CQ are both 4-aminoquinoline antimalarial drugs with very similar chemical structures and similar lysosomotropic properties [28]. Based on the antiviral activities and the mechanism of CQ, it was foreseen that NPQ should have the similar biological effects. The in vitro antimalarial activity of NPQ against Plasmodium falciparum is much better than that of CQ [29]. Their antimalarial activities were closely related to their lysosomotropic properties. In this study, we found that NPQ had similar anti-coronavirus activity to CQ. The experimental results showed that both NPQ and CQ could effectively inhibit SARS-CoV-2 replication when the drugs were added prior to infection or after the initiation and establishment of infection. These results are consistent with a previous study of CQ on SARS-CoV infection of primate cells [30]. The results indicate NPQ may have a similar antiviral mechanism as CQ, which interferes with the terminal glycosylation of the cellular receptor, angiotensin-converting enzyme 2 [30].

Recently, lethal side effects of CQ have raised safety concerns [19], which was also one reason for the cancellation of emergency use. In our experiments, CC_50_s of NPQ for Huh7, H460 and Vero cells were lower than that of CQ, but it is actually different in vivo experiments. It is found that the toxic reactions of beagle dogs caused by po administration of NPQ were much slighter than CQ at the same dose in our lab [31]. The therapeutic window of NPQ was even wider than CQ [31]. In clinical trials, the common clinical side effects of oral NPQ were relatively mild, and did not require treatment [23]. Compared with CQ, there were no severe cardiac side effects related to drugs in tens of thousands of patients treated with NPQ alone, or in combination [23]. Others, such as hypoglycemia, retinal injury, acute extrapyramidal reactions, bone or neuromuscular weakness, and skin disease deterioration, have not been reported. Although animal studies showed that NPQ was slightly toxic to the liver, there was no serious hepatotoxicity except a transient increase in plasma transaminase which could return to normal within 7–14 days [23]. The clinic results indicated that the safety of NPQ was even better than that of CQ.

In addition, NPQ has a long half-life up to 23 days in humans [23,32], and its excellent efficacy for seasonal malaria chemoprophylaxis with monthly single dose has been reported [33,34]. Therefore, NPQ is good for achieving a long-lasting antiviral state by a single oral administration, and it has the potency to be used as a chemoprophylaxis for coronavirus infection. 

In summary, our data provide foundational evidence that proposes that NPQ has broad-spectrum anti-coronavirus properties, and can be used as an alternative drug for coronavirus infection treatment. Its efficacy will be further evaluated in the future through in vivo or clinical testing.

## 4. Materials and Methods

### 4.1. Cell Culture

H460 cells, Huh7 cells, and Vero cells were cultured in Dulbecco’s modified Eagle’s medium (DMEM), supplemented with 10% fetal bovine serum (FBS, Sigma-Aldrich, Shanghai, China) and 1% Pen-strep at 37 °C in 5% CO_2_.

### 4.2. Reagents

NPQ was provided by Sichuan Zihaoshidai Pharmaceuticals Inc. (Chengdu, China), and dissolved in H_2_O. Chloroquine phosphate (Sigma-Aldrich, Shanghai, China, BCBJ1498V) was obtained and dissolved in H_2_O. Ribavirin injection (100 mg/mL) was provided by Hubei Tianyao Pharmaceuticals Inc. (Xiangyang, China, 31712252), and diluted when used.

### 4.3. Viruses

HCoV-229E (strain VR740) was purchased from ATCC. HCoV-OC43 (strain VR1558) was a kind gift from Dr. Xuesen Zhao at Beijing Ditan Hospital, Capital Medical University (Beijing, China). HCoV-229E and HCoV-OC43 propagated on Huh7.5 and HCT-8 cells respectively before they were used. SARS-CoV-2 strain BetaCoV/Beijing/IME-BJ01/2020 was originally isolated from a COVID-19 patient, and the full genome sequence is deposited in the Genome Warehouse in national Genomics Data Center, Beijing Institute of Genomic, CAS, with the accession Nos. GWHACAX01000000. SARS-CoV-2 propagated once on Vero cells before it was used for this study. Studies involving the SARS-CoV-2 were performed at the biosafety level-3laboratory.

### 4.4. Cytotoxicity Measurement

The cytotoxic effects of compounds on H460 cells and Huh7 cells were assayed by CPE assay. Briefly, cells were seeded into 96-well culture plates and were incubated overnight. Then, the medium was removed and different concentrations of compounds were applied in triplicate. After 2 days’ incubation, the cytotoxicity of compounds was determined by CPE assay, and the 50% cytotoxic concentration (CC_50_) was calculated by the Reed–Muench method.

Evaluation of the cytotoxicity of NPQ and CQ on Vero cells were carried out by MTS cell proliferation assays. Briefly, Vero cells were added with different doses of either compounds in triplicate. After 3 days incubation at 37 °C, MTS assays were performed according to the manufacturer’s protocols. After adjusting the absorbance for background (medium) and comparing to untreated controls (untreated cell medium), the cytotoxic concentration CC_50_ was calculated using a sigmoidal nonlinear regression function to fit the dose–response curve using the GraphPad Prism 7.01 software.

### 4.5. CPE Inhibition Assay in H460 and Huh7 Cells

Cells were plated into 96-well cultureplates and incubated for 24 h. The cells were infected with 100 times 50% tissue culture infective dose (TCID_50_) HCoV-229E or HCoV-OC43, and different concentrations of compounds were added simultaneously. HCoV-229E-infected Huh7 cells were treated for about 48 h and HCoV-OC43-infected H460 cells were treated for about 72 h. The 50% inhibition concentration (IC_50_) was determined by Reed–Muench method. The selectivity index(SI) was calculated as the ratio of CC_50_/IC_50_.

### 4.6. Measurement of Viral RNA 

Viral RNA from cell supernatant was extracted by using the QIAamp Viral RNA Mini Kit (Giagen, Cat no. 52904)according to the manufacturer’s instructions. Viral RNA was performed by qRT-PCR using One Step Prime Script RT-PCR Kit (Takara, Japan) with the following primers and probe: CoV-F3 (5′-TCCTGGTGATTCTTCTTCAGGT-3′); CoV-R3 (5′-TCTGAGAGAGGGTCAAGTGC-3′); and CoV-P3 (5′-FAM-AGCTGCAGCACCAGCTGTCCA-BHQ1-3′).

### 4.7. The Anti-SARS-CoV-2 Activity in Vero Cells

The in vitro antiviral efficiency of compounds on Vero cells was determined by qRT-PCR assay. Briefly, Vero cells were pretreated with different dose compounds for 12 h, and then infected with 100 TCID_50_ of SARS-CoV-2 for 1 h at 37 °C. The virus–drug mixture was removed and the cells were further cultured with fresh drug-containing medium. At 48 h, the SARS-CoV-2 RNA copies in supernatant was quantified by qRT-PCR. The IC_50_ was calculated according to the dose–response curves obtained and analyzed using the GraphPad Prism software.

### 4.8. Time-of-Addition Assay

Briefly, Vero cells were infected with 100 TCID_50_ of SARS-CoV-2. NPQ and CQ(10 µM) were added and kept at different time periods: (a) full time throughout the entire assay (whole: 1 h before and 48 h after virus inoculation); (b) only the early phase of the assay (entry: initial 1 h and 2 h after virus inoculation); (c) during the late phase of the assay (post-entry: last 46 h after virus inoculation). All the cells were harvested at 48 h post-infection, and the SARS-CoV-2 RNA copies in supernatant were quantified by qRT-PCR.

## 5. Patents

All of these results have been filed for a Chinese patent. 

## Figures and Tables

**Table 1:** The anti-HCoV-229E activity of NPQ, CQ and RBV in Huh7 cells.

Compounds	CC_50_ (µM)	IC_50_ (µM)	SI
NPQ	11.50 ± 6.19	2.05 ± 1.44	5.61
CQ	71.76 ± 35.59	8.74 ± 7.27	8.21
RBV	409.48 ± 0	15.43 ± 5.57	26.54

**Table 2:** The anti-HCoV-OC43 activity of NPQ, CQ and RBV in H460 cells.

Compounds	CC_50_ (µM)	IC_50_ (µM)	SI
NPQ	>82.52	5.83 ± 0.74	>14.15
CQ	>96.92	3.16 ± 0.60	>30.67
RBV	317.80 ± 0	20.72 ± 7.86	15.34

**Table 3:** The anti- SARS-CoV-2 activity of NPQ and CQ in Vero cells using qRT-PCR.

Compounds	CC_50_ (µM) ^1^	IC_50_ (µM)	SI
NPQ	13.50 ± 0.20	2.01 ± 0.38	6.72
CQ	76.58 ± 0.02	5.85 ± 0.97	13.09

^1^ The cytotoxicity of drugs was carried out by an MTS based assay.

## Data Availability

Not applicable.

## Sample Availability

Samples of the compounds NPQ and CQ are available from the authors.
